# Vitamin D Receptor Gene BsmI Polymorphism in Polish Patients with Systemic Lupus Erythematosus

**DOI:** 10.1155/2013/427818

**Published:** 2013-06-18

**Authors:** Beata Kaleta, Jarosław Bogaczewicz, Ewa Robak, Anna Sysa-Jędrzejowska, Małgorzata Wrzosek, Weronika Szubierajska, Piotr Mróz, Jacek Łukaszkiewicz, Anna Woźniacka

**Affiliations:** ^1^Department of Biochemistry and Clinical Chemistry, Medical University of Warsaw, 02-097 Warsaw, Poland; ^2^Department of Dermatology and Venereology, Medical University of Lodz, 94-017 Lodz, Poland; ^3^Department of Pharmacogenomics, Medical University of Warsaw, 02-097 Warsaw, Poland

## Abstract

The hormonally active form of vitamin D_3_, 1,25(OH)_2_D_3_ (calcitriol), exerts actions through VDR receptor, which acts as a transcriptional factor. Calcitriol is an immunomodulator that affects various immune cells, and several studies link it to many autoimmune diseases. BsmI polymorphism affects the level of *VDR* gene transcription, transcript stability, and posttranscriptional modifications. It seems to be related to the systemic lupus erythematosus (SLE). Our study examined the characteristics of *VDR* gene BsmI polymorphism in Polish SLE patients and their relationship with clinical manifestations of the disease. We genotyped 62 patients with SLE and 100 healthy controls using the real-time PCR. There were no differences observed in the frequency of BsmI genotypes in SLE patients and in the control group. There was no significant correlation between BsmI genotypes and clinical symptoms of SLE, but the AA genotype correlates with higher levels of antinuclear antibodies (ANA) in this group (*r* = 0.438; *P* = 0.002). A larger study examining BsmI and other *VDR* gene polymorphisms is needed. It may allow explaining differences in the clinical picture of the disease and choosing a personalized therapy.

## 1. Introduction

Systemic lupus erythematosus is a chronic antibody-mediated autoimmune disorder. The etiology of SLE is still unknown, but many studies demonstrate association between the disease and genes which are crucial to immunological response [1, 2]. Active form of vitamin D, 1,25(OH)_2_D_3_, exerts action by binding to the VDR (vitamin D receptor) which acts as a ligand-dependent transcriptional factor. VDR are present not only in tissues related to calcium-phosphorus homeostasis (bone, skin, kidneys, and intestine) but also in nonclassical tissues, among others immune cells [3, 4]. The VDR protein is synthesized from a gene known as *VDR* which is highly polymorphic. The most significant polymorphisms for VDR activity are FokI (rs2228570) and BsmI (rs1544410). BsmI polymorphism is located in intron 8 and affects the level of *VDR* gene transcription, transcript stability, and posttranscriptional modifications [5–10]. VDR are present in nearly all immune cells. 1,25(OH)_2_D_3_ blocks B cell differentiation and proliferation, enhances chemotactic and phagocytotic capacity of macrophages, inhibits DC maturation, and modulates DC-derived cytokine and chemokine expression, by inhibiting production of IL-12, IL-23 and enhancing release of IL-10. In addition vitamin D inhibits the surface expression of MHC-II-complexed antigen and costimulatory molecules, affects T cells response, inhibits production of Th1 cytokines (IL-2, IF-*γ*), Th17 cytokines (IL-17, IL-21), and stimulates Th2 cytokine production (IL-4). Moreover this hormone reduces Th induction of immunoglobulin (IgM, IgG) production by B cells [11–21]. Moreover immune cells express enzymes, that is, 25-hydroxylase, 1 *α*-hydroxylase, which are needed to generate bioactive 1,25(OH)_2_D_3_ [22, 23]. All of this suggest that 1,25(OH)_2_D_3_ plays a key role in immune homeostasis. It is known that the deficiency of active vitamin D and polymorphism of the vitamin D receptor (VDR) are associated with increased incidence of several autoimmune diseases [24–40]. An association between *VDR* gene polymorphisms and systemic lupus erythematosus in Asian patients has been reported [1, 2, 34, 41, 42].

As the literature data indicates differences in the distribution of BsmI genotypes between Chinese and European population, our study was conducted in order to evaluate relationship between this polymorphism and clinical and laboratory profiles in Polish patients with SLE.

## 2. Materials and Methods

The study involved 62 Polish patients (57 women, 5 men) with SLE treated at the Department of Dermatology and Venereology, Medical University of Łodz, Poland. All patients fulfilled at least four out of eleven criteria for SLE classification [43]. This group was selected randomly. 100 healthy subjects (63 women, 37 men) served as controls. They did not meet criteria for SLE and other autoimmune diseases. Short characteristic of SLE patients and control subjects is presented in Table 1.

Genomic DNA was extracted from peripheral full blood using “Blood Mini” kit from A&A Biotechnology and following the protocol of producer. VDR BsmI genotyping was performed by real-time polymerase chain reaction (RT-PCR, LightCycler, Roche) with SimpleProbe (TIB MOLBIOL) melting-curve analysis in accordance with the conditions showed in Table 2.

It enables to identify individual BsmI genotypes (polymorphic variants) of vitamin D receptor gene. The genotypes were classified as homozygote major allele (GG), heterozygote (GA) and homozygote minor alleles (AA).

Statistical analyses were performed using Statistica 10.0 (StatSoft Inc.). To compare the frequency of genotypes and alleles of VDR BsmI polymorphism in patients with SLE and control group, the Freeman-Halton extension of Fisher's exact test and Fisher's exact test were used. Correlation analysis of BsmI genotypes with clinical manifestations and laboratory profiles of SLE was performed using Spearman's Rank Correlation Test. Hardy-Weinberg equilibrium (HWE) was determined by Pearson's *χ* ^2^ goodness-of-fit test. Differences were considered statistically significant at a *P* value <0.05.

The study was approved by the Local Ethics Committee (no. RNN/67/08/KE).

## 3. Results and Discussion

Table 3 presents VDR BsmI genotypes and alleles in patients with SLE and in control group. The distribution of genotypes was 53% for GG, 32% for GA, and 14% for AA in patients with SLE and, respectively, 41%, 42%, and 17% in control group. There was no statistically significant difference between these groups (*P* = 0.309). The allelic distribution of G and A was similar within the two groups (*P* = 0.188). The genotype frequencies were consistent with HWE in patients and controls (*χ* ^2^ = 3.60; *P* = 0.058 and *χ* ^2^ = 1.18; *P* = 0.277, resp.).

The relationship between VDR BsmI genotypes and clinical manifestation or laboratory profiles of SLE is demonstrated in Table 4. There is no relationship between BsmI genotypes and clinical symptoms of SLE, but it was shown that AA genotype of BsmI polymorphism is in correlation with higher titer of antinuclear antibodies' (*r* = 0.438; *P* = 0.002) (Table 5).

Vitamin D exerts several immunomodulatory effects and thus may play a role in the course of autoimmune diseases. Its active hormonal form 1,25(OH)_2_D_3_, among others, blocks B cell proliferation, differentiation, and immunoglobulin production [7–13].

Numerous studies have shown that vitamin D deficiency may increase the risk of autoimmune diseases such as systemic lupus erythematosus [16–32]. The role of vitamin D receptor gene polymorphism can modify the immunomodulatory action of vitamin D and has an impact on the clinical picture of SLE. It has been demonstrated that *VDR* gene BsmI polymorphism is a genetic marker of systemic lupus erythematosus in Asian patients [1, 2, 40–42]. In our study we analyzed vitamin D receptor gene BsmI polymorphism in the Polish patients with SLE. We did not find statistically significant differences in the frequency of BsmI genotypes and alleles in SLE patients and healthy controls. Our observations are consistent with the findings of Mostowska et al. [44], Monticielo et al. [45], and Sakulpipatsin et al. [46] who demonstrated no association of the BsmI polymorphism with the development of SLE in the Brazilian, Iranian, Thai, and European populations. However, this polymorphism was associated with lupus nephritis in a Japanese population [41]. Different results of various studies may be due to group size, gene–gene interactions, and environmental factors. Our study showed that AA genotype of BsmI polymorphism is associated with higher concentration of antinuclear antibodies (ANA) in patients with SLE. However it is difficult to explain this phenomenon as it is generally known that ANA titer does not correlate with the disease activity and is not considered as a valuable prognostic factor. It is possible that BsmI polymorphism changes the immunomodulatory action of 1,25(OH)_2_D_3_ resulting in modified antibodies production by activated B cells.

## 4. Conclusions

The BsmI genotype frequencies in Polish patients with SLE are not different from healthy controls. There is no significant correlation of this polymorphism and clinical symptoms of systemic lupus erythematosus. Our data did not reveal that in Polish patients with SLE *VDR* gene BsmI polymorphism could be regarded as a genetic marker of the disease. AA genotype of BsmI polymorphism promotes a higher concentration of ANA in SLE, patients but future studies are needed to confirm the validity of these observations.

## Figures and Tables

**Table 1:** Characteristic of SLE patients and control subjects.

	SLE	Control
Number of subjects	62	100
Age (year)	27–79 (49)	18–71 (39)
Female/male	57/5	63/37
ANA/l	320–2560 (1560)	NA

**Table 2:** Real-time PCR reaction conditions.

Parameter	Program							
	Denat.	Cycling			Melting			Cooling
Analysis mode	None	Quantification			Melting curves			None
Cycles	1	45			1			1
Segment	1	1	2	3	1	2	3	1
Target [°C]	95	95	60	72	95	40	75	40
Hold [hh:mm:ss]	00:10:00	00:00:10	00:00:10	00:00:15	00:00:30	00:02:00	00:00:00	00:00:30
Ramp rate [°C/s]	4.4	4.4	2.2	4.4	4.4	1.5	—	1.5
Acquisition mode	None	None	Single	None	None	None	Continu.	None
Acquisition per [°C]							3	

**Table 3:** Distribution of VDR BsmI genotypes and alleles in patients with SLE and healthy controls.

BsmI (rs1544410) polymorphism of *VDR* gene					
Group	Genotype (%)			Alleles	
	GG (wt)	GA	AA (mt)	G	A
SLE^1^ (*n* = 62)	33 (53)	20 (32)	9 (14)	86 (0.7)	38 (0.3)
Control^2^ (*n* = 100)	41 (41)	42 (42)	17 (17)	123 (0.6)	77 (0.4)
Statistics*	*χ* ^2^ = 2.35; df = 2; *P* = 0.309			*χ* ^2^ = 1.734; df = 1; *P* = 0.188	

*Freeman-Halton extension of Fisher's exact test and Fisher's exact test.

^1^HWE: *χ* ^2^ = 3.60; df = 1; *P* = 0.058.

^2^HWE: *χ* ^2^ = 1.18; df = 1; *P* = 0.277.

**Table 4:** Relationship between BsmI genotypes and clinical manifestation or laboratory profiles of SLE.

	GG *n* = 33	GA *n* = 20	AA *n* = 9	Total *N* = 62	P*
Malar rash	14/33	13/20	6/9	33/62	0.216
Discoid rash (DLE)	4/33	1/20	0/9	5/62	0.549
Photosensitivity	6/33	0/20	3/9	9/62	0.145
Oral ulcer	5/33	0/20	1/9	6/62	0.171
Arthritis/arthralgia	30/33	18/20	9/9	57/62	1.000
Serositis	3/33	1/20	1/9	5/62	0.840
Renal disorder	5/33	2/20	1/9	8/62	0.876
Neuropsychiatric disorder	7/33	4/20	2/9	13/62	1.000
Hematologic disorder					
(i) Anemia	9/33	2/20	2/9	13/62	0.348
(ii) Leukopenia	16/33	6/20	6/9	28/62	0.172
(iii) Lymphopenia	1/33	1/20	0/9	2/62	1.000
(iv) Thrombocytopenia	9/33	2/20	0/9	11/62	0.135
Immunologic disorder					
(i) Anti-dsDNA	9/33	1/20	2/9	12/62	0.124
(ii) Anti-Sm	0/33	0/20	0/9	0/62	1.000
ANA presence	29/33	18/20	8/9	55/62	1.000

*Freeman-Halton extension of Fisher's exact test and Fisher's exact test.

**Table 5:** Correlation between BsmI genotypes and higher titer of antinuclear antibodies in SLE patients.

Correlated variables*	
BsmI genotype versus ANA/L	*r* = 0.438
	*P* = 0.002

*Spearman's Rank Correlation Test, BsmI genotypes (ranks): 0: GG, 1: GA, and 2: AA; ANA/L: antinuclear antibodies per liter; *r*: Spearman's rank correlation coefficient.

## Abbreviations

ANA: Antinuclear antibodies
DC: Dendritic cells
HWE: Hardy-Weinberg equilibrium
IF: Interferon
IgG: Immunoglobulin G
IgM: Immunoglobulin M
IL: Interleukin
MHC: Major histocompatibility complex
RT-PCR: Real-time polymerase chain reaction
SLE: Systemic lupus erythematosus
Th: T helper cell
VD: Vitamin D
VDR: Vitamin D receptor.
